# Liver–metabolic stress, apolipoprotein E ε4, and cognition and amyloid burden: findings from the dementia platform Korea trial-ready registry

**DOI:** 10.3389/fnagi.2026.1773977

**Published:** 2026-03-11

**Authors:** Young Hyeon Ahn, Jin Gu Kang, Dahyeon Choi, Dae-Jin Kim, Chan-Mo Yang, Sang-Yeol Lee, Sae Hwan Lee, Young Hyun Jung, Sung-Hoon Yoon

**Affiliations:** 1 Department of Gastroenterology, Soonchunhyang University Cheonan Hospital, Cheonan, Republic of Korea; 2 Department of Psychiatry, Wonkwang University Hospital, Iksan, Republic of Korea; 3 Department of Psychiatry, School of Medicine, Wonkwang University, Iksan, Republic of Korea; 4 Department of Physiology, College of Medicine, Soonchunhyang University, Cheonan, Republic of Korea; 5 Institute for Molecular Metabolism Innovation, Soonchunhyang University, Asan, Republic of Korea

**Keywords:** amyloid, brain–liver–metabolic axis, dementia, fibrosis-4 index, insulin resistance, liver fibrosis, triglyceride–glucose index

## Abstract

**Introduction:**

Liver–metabolic stress and apolipoprotein E (APOE) ε4 are implicated in late-life cognitive vulnerability, yet how hepatic–metabolic indices relate to cognition and amyloid burden and whether these associations vary by APOE ε4 allele dose remains unclear. We examined liver–metabolic indices in relation to cognition and amyloid PET SUVR and tested effect modification by APOE ε4.

**Methods:**

We analyzed baseline data from the Dementia Platform Korea Trial-Ready Registry (DPK-TRR). Primary multivariable analyses used complete cases for outcomes and covariates (*n* = 507); amyloid PET analyses used the PET subset (*n* = 496). Exposures included the triglyceride–glucose (TyG) index, AST/ALT ratio, and Fibrosis-4 (FIB-4) stage (low/intermediate/high). APOE ε4 was modeled as allele dose (0/1/2). Multivariable linear regression evaluated associations with global cognition (MMSE), selected domain outcomes, and amyloid PET SUVR, including FIB-4 stage × APOE ε4 dose interaction terms, adjusted for age, sex, education, hypertension, diabetes, and dyslipidemia.

**Results:**

Higher TyG index and AST/ALT ratio were associated with lower MMSE scores (TyG: *β* = −1.13, 95% CI − 2.11 to −0.16; *p* = 0.022; AST/ALT: *β* = −1.40, 95% CI − 2.10 to −0.39; *p* = 0.007) and with memory performance on CERAD delayed recall (TyG: *β* = −0.29, 95% CI − 0.52 to −0.06; *p* = 0.012), whereas neither marker showed a clear association with amyloid PET SUVR. The association between FIB-4 stage and cognition differed by APOE ε4, showing a cross-over pattern across fibrosis stages: APOE ε4–associated differences were attenuated and reversed at intermediate/high FIB-4 compared with low FIB-4. In the amyloid PET subset, APOE ε4 group differences in SUVR were not prominent at low FIB-4, but tended to diverge at high FIB-4 with higher SUVR in groups with greater ε4 burden.

**Conclusion:**

Routine liver–metabolic indices were associated with cognitive performance, while FIB-4 stage showed effect modification by APOE ε4 in relation to both cognition and amyloid PET SUVR. These findings support heterogeneity in liver–metabolic and genetic contributions to late-life cognitive vulnerability in a dementia trial-ready registry and motivate longitudinal studies to clarify temporal relationships.

## 1 Introduction

Late-life dementia arises from the convergence of neurodegenerative, vascular, and systemic processes that accumulate over decades, with Alzheimer’s disease (AD) pathology representing only one component within this broader etiological landscape (Livingston et al., 2020; Jack Jr et al., 2018). AD is biologically defined by the cerebral *β*-amyloid and tau deposition, which together constitute the neuropathological core of the disease (Long and Holtzman, 2019). Among genetic risk factors, the apolipoprotein E (APOE) ε4 allele is the strongest and most consistent determinant of late-onset AD risk, largely through its effects on *β*-amyloid aggregation and clearance (Verghese et al., 2013; Liu et al., 2017). However, accumulating evidence indicates that vascular and metabolic factors such as insulin resistance, dyslipidemia, and metabolic dysfunction–associated steatotic liver disease (MASLD) can influence the risk, timing, and clinical expression of AD by modulating amyloid and tau pathology or by lowering the threshold for cognitive impairment (Zhang J. et al., 2024; Poddar et al., 2021; Lim et al., 2026). These overlapping pathophysiological processes may jointly erode cognitive reserve and accelerate the transition from preclinical pathology to overt dementia in susceptible individuals.

Epidemiological and biomarker studies increasingly demonstrate that systemic metabolic and hepatic abnormalities are associated with late-life cognitive decline and dementia, beyond classical Alzheimer pathology (Vegas-Suarez et al., 2022; Liu X. L. et al., 2024). Triglyceride–glucose (TyG) index, a surrogate marker of insulin resistance, has been linked to poorer cognitive performance and an increased risk of mild cognitive impairment (MCI) in meta-analytic studies (Liu Y. et al., 2024). In parallel, MASLD and greater liver fibrosis burden, as indexed by noninvasive measures such as the Fibrosis-4 index (FIB-4) score, have been associated with brain atrophy, cerebral small-vessel disease, and amyloid-related pathologies, which have been linked to elevated dementia and AD risk (Fan et al., 2025; Wang et al., 2026; Nagaoka et al., 2025; Weinstein et al., 2022). Notably, APOE ε4 extends beyond its canonical role in amyloid processing to serve as a key regulator of peripheral lipid transport and hepatic fat metabolism (Jun, 2025). Several studies further suggest that metabolic syndrome or insulin resistance exerts a greater adverse cognitive impact in APOE ε4 carriers than in non-carriers (Jabeen et al., 2022; Padovani et al., 2025). Collectively, these findings support the emerging concept of a brain–liver–metabolic axis and raise the hypothesis that liver–metabolic stress and APOE ε4 may interact to shape Alzheimer-related cognitive trajectories and amyloid mechanisms, although the specific pathways remain incompletely understood.

Mechanistic evidence further supports this brain–liver–metabolic axis concept. The liver is a central regulator of systemic metabolism, and hepatic steatosis and fibrosis can promote insulin resistance, chronic low-grade inflammation, and atherogenic dyslipidemia, thereby creating a biological milieu permissive to neurodegenerative change (Zeng and Lo, 2025). In parallel, impaired hepatic function may reduce the peripheral clearance of circulating amyloid-*β* and other neurotoxic metabolites, indirectly facilitating their accumulation in the brain (Peng et al., 2024; Darabi et al., 2025). Experimental and clinical studies of MASLD and elevated liver fibrosis indices, including FIB-4 score, have linked liver–metabolic stress to increased dementia risk, AD-related brain changes, and poorer late-life cognitive performance (Shang et al., 2022; Parikh et al., 2022). Together, these observations suggest that liver–metabolic stress may lower the threshold at which age and APOE ε4–related AD pathology translates into measurable cognitive impairment, providing a rationale for investigating liver-derived markers alongside APOE ε4, cognition, and amyloid burden within the same cohort.

Against this background, most prior studies have examined associations among insulin resistance, liver fibrosis, cognition, or Alzheimer-related biomarkers in isolation (Liu et al., 2024; Fan et al., 2025; Wang et al., 2026). Few investigations have evaluated liver–metabolic stress markers (including the TyG index, AST/ALT ratio, and FIB-4), detailed domain-specific cognitive performance, and amyloid PET burden within a single cohort using a consistent analytical framework. Moreover, the potential modifying role of APOE ε4 in these associations has not been fully characterized (Nagaoka et al., 2025; Weinstein et al., 2022). Whether liver–metabolic stress interacts with APOE ε4 allele burden to influence both cognitive function and cortical amyloid deposition in late life therefore remains unclear. Accordingly, using data from the Dementia Platform Korea Trial-Ready Registry (DPK-TRR), we aimed to (1) examine the associations of the TyG index, AST/ALT ratio, and FIB-4 stage with global and domain-specific cognitive performance; (2) test whether FIB-4 stage interacts with APOE ε4 allele dose (0/1/2) in relation to cognition; and (3) in the amyloid PET subset, evaluate whether FIB-4 stage and its interaction with APOE ε4 allele dose are associated with cortical amyloid burden.

## 2 Materials and methods

### 2.1 Study population: dementia platform Korea trial-ready registry cohort

This study used cross-sectional baseline data from the DPK-TRR cohort (Kim et al., 2023). Participants were drawn from the DPK-TRR, an ongoing multicenter cohort designed to enroll older adults across the cognitive spectrum and to harmonize clinical, neuropsychological, imaging, and biomarker data for dementia-related research. Individuals aged ≥ 40 years who completed comprehensive baseline evaluations—including clinical assessments, laboratory testing with liver and metabolic markers, brain magnetic resonance imaging (MRI), and neuropsychological assessment—were eligible for inclusion.

**Figure 1:**
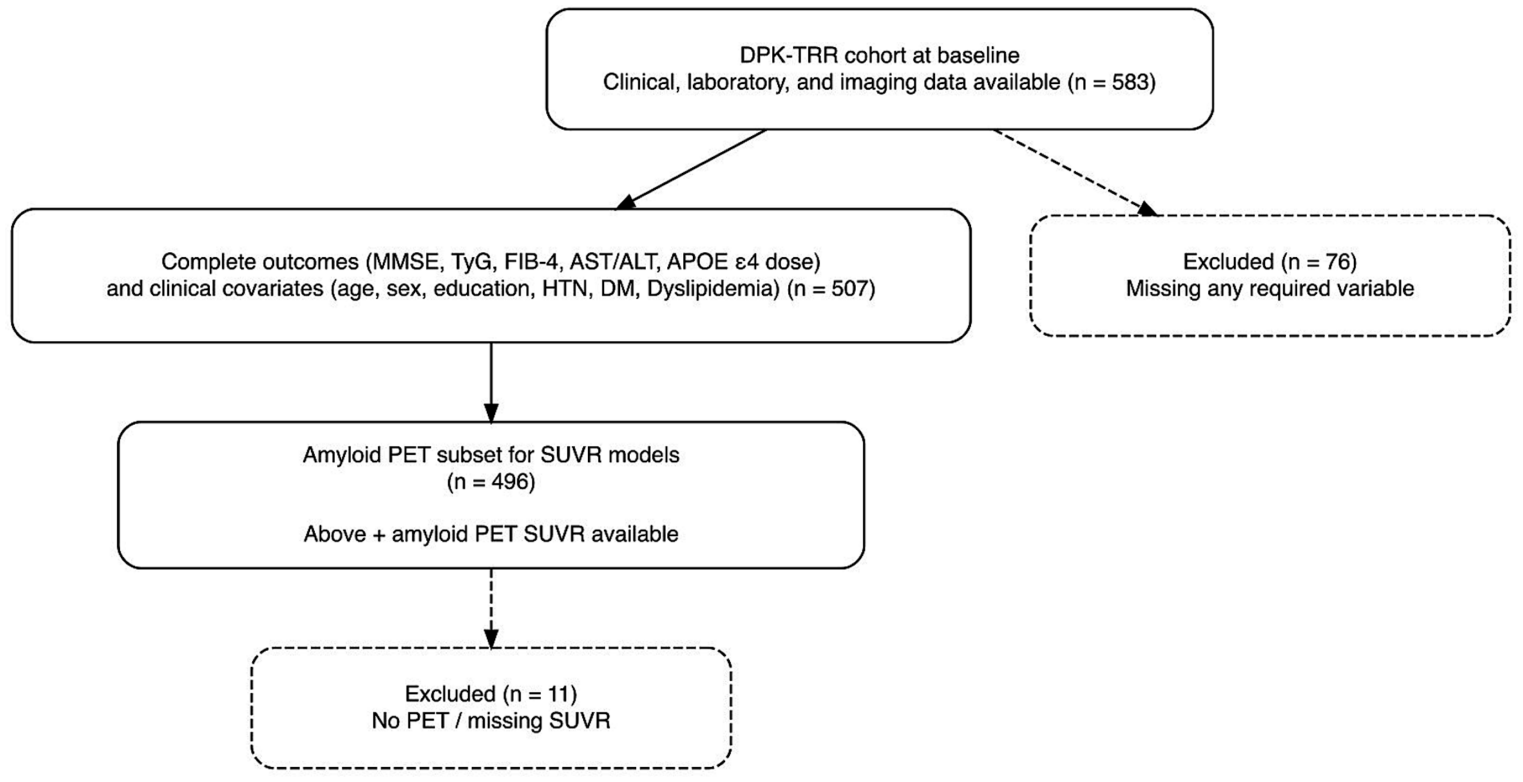
Flow diagram of study population selection.

From the DPK-TRR baseline cohort, we identified 583 participants with available baseline neuropsychological data, fasting laboratory assessments of liver and metabolic markers, APOE genotyping, and structural MRI. For the primary multivariable analyses, we used a complete-case sample with non-missing outcomes and covariates (*n* = 507). Among these participants, those who additionally underwent amyloid PET imaging (*n* = 496) were included in the analyses of cortical amyloid burden. Participants with missing liver–metabolic markers, incomplete cognitive data, missing APOE ε4 genotype, or unavailable amyloid PET measures were excluded from the corresponding analyses (Figure 1).

All participants provided written informed consent in accordance with the Declaration of Helsinki. The DPK-TRR protocol was approved by the Institutional Review Board (IRB No. 2020–10-077-035/B-2011-651-001), and this secondary analysis was approved by the Institutional Review Board of Wonkwang University Hospital (IRB No. WKUH 2025–12-024).

### 2.2 Clinical and demographic assessment

All participants underwent standardized clinical assessments at the baseline DPK-TRR visit. Demographic information included age, sex, and years of formal education, obtained through structured interviews.

Vascular and metabolic comorbidities were documented based on physician diagnosis and current medication use. Hypertension, diabetes mellitus, and dyslipidemia were each coded as present or absent according to the interviews and the use of antihypertensive, antidiabetic, or lipid-lowering medications. These conditions were treated as covariates in multivariable analyses to account for potential confounding effects of vascular and metabolic risk on cognitive performance and amyloid burden.

Global cognitive status was evaluated using the Mini-Mental State Examination (MMSE), administered by trained clinicians or psychometrics’ according to standardized procedures. The total MMSE score served as the primary index of global cognitive function, with higher scores indicating better performance. Domain-specific cognitive measures derived from comprehensive neuropsychological batteries are described in the following section.

### 2.3 Liver–metabolic indices

Fasting venous blood samples were collected in the morning after an overnight fast and analyzed at certified laboratories using standardized automated assays. For this study, we examined three complementary liver–metabolic indices: the TyG index, AST/ALT ratio, and FIB-4 index.

The TyG index was calculated as a surrogate marker of insulin resistance using the following formula:

$$\begin{array}{l} \mathrm{TyG}\;\text{index}=\\ \ln(\frac{\text{fasting triglycerides}\;(\mathrm{mg}/\mathrm{dL})\times \text{fasting glucose}\;(\mathrm{mg}/\mathrm{dL})}{2}) \end{array}$$

and was analyzed as a continuous variable (Simental-Mendía and Guerrero-Romero, 2020).

The AST/ALT ratio was defined as the serum concentration of AST divided by ALT. This ratio was used as an indicator of hepatocellular injury pattern and liver–metabolic stress and was treated as a continuous variable in the regression models (Sorbi et al., 1999).

The FIB-4 index was used as a noninvasive marker of liver fibrosis and was calculated according to the standard formula (Sterling et al., 2006):


$$\mathrm{FIB}-4=\frac{\mathrm{age}\;(\text{years})\times \mathrm{AST}\;(\mathrm{IU}/L)}{\text{platelet count}(10^{9}/L)\times \sqrt{\mathrm{ALT}\;(\mathrm{IU}/L)}}.$$

In addition to analyses using FIB-4 as a continuous measure, participants were categorized into three FIB-4 stages based on established cutoffs (Shah et al., 2009):

- Low: FIB-4 < 1.3
- Intermediate: 1.3 ≤ FIB-4 < 2.67
- High: FIB-4 ≥ 2.67

These categories were used to represent increasing liver fibrosis burden and to examine potential interaction effects with APOE ε4 carrier status on cognitive performance and amyloid burden.

Collectively, these indices were selected to capture key aspects of liver–metabolic stress, including insulin resistance (TyG index), hepatocellular injury pattern (AST/ALT ratio), and liver fibrosis (FIB-4 index).

### 2.4 Neuropsychological assessment

Neuropsychological testing was conducted following a standardized protocol using validated Korean versions of established cognitive batteries. All assessments were administered by trained neuropsychologists or psychometrics’ under the supervision of dementia specialists.

Global cognitive function was assessed using the Korean version of the MMSE (K-MMSE), which has been adapted and standardized from the original MMSE for use in older Korean populations (Folstein et al., 1975; Kang, 2006). The total K-MMSE score served as the primary index of global cognitive function, with higher scores indicating better performance.

For domain-specific cognitive assessment, we used the Seoul Neuropsychological Screening Battery–II (SNSB-II) and the Korean version of the Consortium to Establish a Registry for Alzheimer’s disease neuropsychological battery (CERAD-K). The SNSB-II is a comprehensive battery that provides demographically adjusted *z*-scores across five cognitive domains—attention, language, visuospatial function, memory, and frontal/executive function—based on large normative datasets from the Korean population (Ryu and Yang, 2023). From the SNSB-II, we selected *a priori* the memory, visuospatial, and frontal/executive domains as primary outcomes because of their relevance to AD and vascular/metabolic cognitive impairment, as well as their demonstrated robust associations with clinical and functional outcomes in prior studies (Park et al., 2011; Yates et al., 2012). Domain scores were analyzed as demographically adjusted z-scores, with higher values indicating better performance.

Additionally, participants completed the CERAD-K neuropsychological battery (Lee et al., 2002), which provides demographically adjusted norms for multiple subtests commonly used in dementia research. From CERAD-K, we derived z-scores for three subtests selected to correspond to the SNSB-II domains of interest. Constructional praxis served as a complementary measure of visuospatial/visuoconstructive function, and the word list delayed recall score was used as a representative index of verbal episodic memory. Both measures are highly sensitive to Alzheimer-related pathology, discriminate MCI from dementia, and predict progression (Fillenbaum et al., 2011; Karrasch et al., 2005; Galvin et al., 2007). Furthermore, the semantic category verbal fluency test was used as an index of frontal/executive–language functioning, as category fluency tasks require self-initiated lexical retrieval, strategic search, and mental set shifting and are considered sensitive to frontal and executive dysfunction as well as semantic memory impairment in aging and dementia (Tombaugh et al., 1999; Oh et al., 2019; Kwak et al., 2022).

In this study, CERAD-K constructional praxis and word list delayed recall *z*-scores were treated as parallel measures of visuospatial and episodic memory, respectively, to the corresponding SNSB-II domain scores. This approach enabled the examination of convergent, domain-specific effects of liver–metabolic markers and APOE ε4 status across two widely used neuropsychological batteries. Recent validation studies have similarly mapped CERAD-K constructional praxis and word list memory/recall tasks onto visuospatial and episodic memory domains when comparing CERAD-K and SNSB-II with newer digital cognitive assessments (Na et al., 2024; Moon et al., 2024).

### 2.5 APOE genotyping and amyloid PET

APOE genotyping was performed using standard polymerase chain reaction–based methods, and APOE ε4 was coded as allele dose (0/1/2; 0 = non-carrier, 1 = heterozygote, 2 = homozygote) for the main analyses. In a subset of participants, amyloid PET imaging was conducted using either ^18^F-florbetaben or ^18^F-flutemetamol, according to the registry imaging protocol and tracer availability at each site. Cortical amyloid burden was quantified as a global standardized uptake value ratio (SUVR), calculated as the mean tracer uptake in a predefined composite cortical region of interest divided by uptake in a reference region. Amyloid SUVR was analyzed as a continuous variable.

### 2.6 Covariates

Demographic covariates included age, sex, and years of education. Vascular risk factors—hypertension, diabetes mellitus, and dyslipidemia—were coded as binary variables based on medical history, medication use, and/or clinical records. These covariates were selected *a priori* as potential confounders of the relationships between liver–metabolic indices, cognition, and amyloid burden (Livingston et al., 2020).

### 2.7 Statistical analysis

Baseline characteristics were summarized according to FIB-4 staging (low, intermediate, or high). Continuous variables were reported as means and standard deviations or medians and interquartile ranges, as appropriate, and compared using analysis of variance or Kruskal–Wallis tests. Categorical variables were summarized as counts and percentages and compared using *χ*^2^ tests or Fisher’s exact tests.

For the primary analyses, multivariable linear regression models were fitted for global cognition (MMSE total score) and selected domain-specific z-scores (SNSB-II visuospatial z and CERAD visuospatial *z*). The primary predictors were FIB-4 stage (low/intermediate/high; low as reference), APOE ε4 dose (0/1/2), and their interaction term (FIB-4 stage × APOE ε4 dose) to test effect modification. Models were adjusted for age_at_dx, sex, years of education, hypertension, diabetes, and dyslipidemia. TyG index and AST/ALT ratio were analyzed as continuous variables in separate models; mean-centering was applied where interaction terms were evaluated. In the amyloid PET subset, analogous multivariable linear regression models were fitted with global cortical amyloid SUVR as the dependent variable. The same set of predictors (TyG index, AST/ALT ratio, FIB-4 staging, APOE ε4 status, and the FIB-4 × APOE ε4 interaction) and covariates (age, sex, years of education, hypertension, diabetes, dyslipidemia) were included, and amyloid SUVR was analyzed as a continuous outcome.

In the amyloid PET subset (*n* = 496), analogous multivariable linear regression models were fitted with global cortical amyloid PET SUVR as the dependent variable. Predictors included FIB-4 stage, APOE ε4 dose, and their interaction, with the same covariate adjustment set.

To aid interpretation of interaction effects, adjusted estimated marginal means (EMMs) of cognitive outcomes and amyloid PET SUVR were derived across FIB-4 stages by APOE ε4 dose from the fitted models and plotted with 95% confidence intervals.

All analyses were conducted using complete cases (*n* = 507 for cognitive analyses; *n* = 496 for amyloid PET analyses). Two-sided *p*-values < 0.05 were considered statistically significant. Given the *a priori* selection of a limited number of cognitive domains and the primarily hypothesis-driven focus on liver–metabolic markers and FIB-4 × APOE ε4 interactions, formal correction for multiple comparisons was not applied; domain-specific results are therefore interpreted as exploratory and in the context of overall patterns. Statistical analyses and figures were generated using R (version 4.5.0; R Foundation for Statistical Computing, Vienna, Austria).

## 3 Results

### 3.1 Participant characteristics

A total of 583 participants had available baseline clinical, laboratory, APOE genotyping, MRI, and neuropsychological data; the primary multivariable analyses were conducted in the complete-case analytic sample (*n* = 507) (Table 1; Figure 1). In the available baseline sample, 307 (53%) participants had dementia, 214 (37%) had MCI, and 62 (11%) were cognitively normal. Participants with dementia were older than those with MCI or normal cognition, whereas years of education and sex distribution were broadly similar across diagnostic categories (Table 1). Vascular risk factors (hypertension, diabetes, and dyslipidemia) were comparable across groups, with modest between-group differences in dyslipidemia prevalence. As expected, global cognitive performance differed substantially by diagnosis.

**Table 1:** Baseline characteristics by cognitive status (normal/mild cognitive impairment/Dementia).

Characteristic	Overall *N* = 507^1^	Normal *N* = 54^1^	MCI *N* = 168^1^	Dementia *N* = 285^1^	*p*-value^2^
Age (years)	72.88 (8.27)	72.06 (7.92)	71.28 (8.22)	73.97 (8.23)	0.001
Education (years)	10.37 (4.80)	9.80 (4.17)	11.05 (4.60)	10.07 (4.99)	0.064
Sex					0.6
Male	176 (35%)	17 (31%)	63 (38%)	96 (34%)	
Female	331 (65%)	37 (69%)	105 (63%)	189 (66%)	
Hypertension	212 (42%)	25 (46%)	66 (39%)	121 (42%)	0.6
Diabetic Mellitus	127 (25%)	9 (17%)	45 (27%)	73 (26%)	0.3
Hyperlipidemia	217 (43%)	26 (48%)	82 (49%)	109 (38%)	0.063
APOE ε4 dose					0.001
Non-carrier (e3/e3)	255 (50%)	40 (74%)	72 (43%)	143 (50%)	
Heterozygote (e3/e4)	184 (36%)	10 (19%)	75 (45%)	99 (35%)	
Homozygote (e4/e4)	68 (13%)	4 (7.4%)	21 (13%)	43 (15%)	
TyG index	8.63 (0.52)	8.52 (0.55)	8.62 (0.49)	8.66 (0.52)	0.2
AST: ALT ratio	1.41 (0.46)	1.34 (0.35)	1.32 (0.37)	1.48 (0.51)	0.009
FIB-4 stage					0.4
Low	79 (16%)	9 (17%)	30 (18%)	40 (14%)	
Intermediate	319 (63%)	31 (57%)	110 (65%)	178 (62%)	
High	109 (21%)	14 (26%)	28 (17%)	67 (24%)	

^1^Mean (SD); *n* (%).

^2^Kruskal-Wallis rank sum test; Pearson’s Chi-squared test.

*Post-hoc* pairwise comparisons with Holm adjustment (chi-square or Fisher’s exact tests for categorical variables) revealed significant differences in APOE ε4 dose distribution between cognitively normal and MCI groups (adjusted *p* = 0.0002) and between cognitively normal and dementia groups (adjusted *p* = 0.001); no other variables showed significant pairwise differences after correction.

SD, standard deviation; MCI, mild cognitive impairment; APOE ε4, apolipoprotein E ε4 allele; TyG index, triglyceride–glucose index; AST/ALT, aspartate aminotransferase/alanine aminotransferase; MMSE, Mini-Mental State Examination; FIB-4, Fibrosis-4 index.

APOE ε4 was coded as allele dose (0/1/2), and its distribution varied across diagnostic categories (Table 1). Liver–metabolic indices showed modest differences by diagnosis, including higher AST/ALT ratio in dementia. The distribution of FIB-4 stages showed a modest shift toward higher categories in dementia, although between-group differences were not pronounced (Table 1).

### 3.2 Associations of triglyceride–glucose index and aminotransferase/alanine aminotransferase ratio with global cognition, memory, and amyloid burden

In multivariable linear regression models adjusted for age, sex, education, hypertension, diabetes, and dyslipidemia, higher liver–metabolic stress indexed by the TyG index and AST/ALT ratio was associated with poorer global cognition (Table 2). Specifically, each one-unit increase in TyG was associated with a 1.13-point lower MMSE score (*β* = −1.13; 95% CI, −2.11 to −0.16; *p* = 0.022), and a higher AST/ALT ratio was also associated with lower MMSE scores (*β* = −1.40; 95% CI, −2.10 to −0.39; *p* = 0.007).

**Table 2:** Associations of TyG index and AST: ALT ratio with global cognition, memory domains, and amyloid burden.

Characteristic	MMSE global			SNSB-II memory z			CERAD delayed recall z			Amyloid PET SUVR		
	*β* (95% CI)	95% CI	*p*-value	*β* (95% CI)	95% CI	*p*-value	*β* (95% CI)	95% CI	*p*-value	*β* (95% CI)	95% CI	*p*-value
TyG	−1.13	−2.11, −0.16	0.022*	−0.31	−0.68, −0.05	**0.087**	−0.29	−0.52, −0.06	0.012*	−0.03	−0.09, 0.03	0.3
AST: ALT ratio	−1.4	−2.1, −0.39	0.007*	−0.37	−0.83, 0.09	0.113	0.00	−0.21, 0.22	0.98	−0.02	−0.08, 0.04	0.57

CI, Confidence interval.

**Figure 2:**
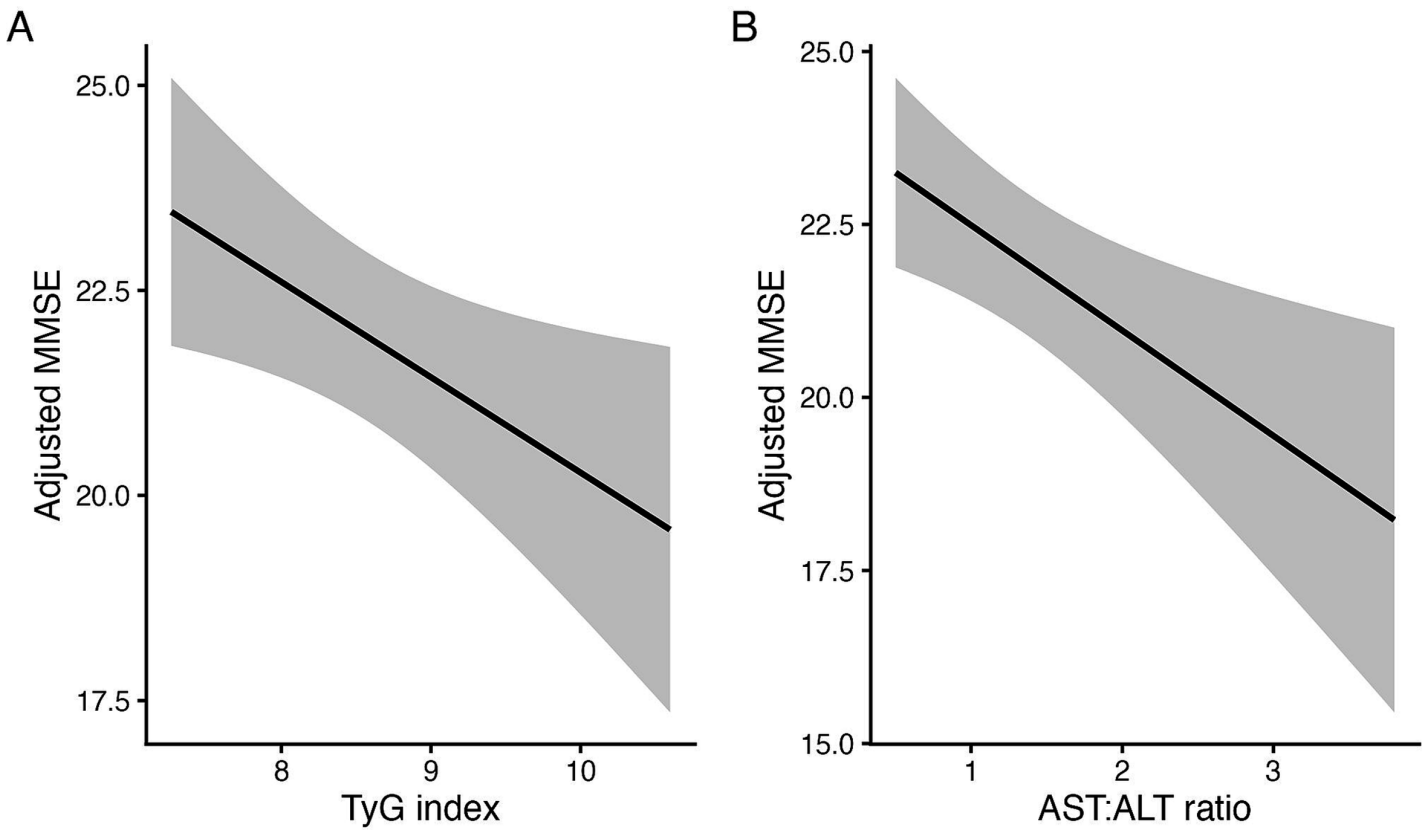
Associations of liver–metabolic stress markers with global cognitive function. Partial effect plots illustrating the adjusted associations of the TyG index (left) and AST/ALT ratio (right) with MMSE scores from multivariable linear regression models. Solid lines indicate model-predicted values and shaded areas indicate 95% confidence intervals. Models were adjusted for age_at_dx, sex, education, hypertension, diabetes, and dyslipidemia. TyG, triglyceride–glucose; AST, aspartate aminotransferase; ALT, alanine aminotransferase; MMSE, Mini-Mental State Examination.

For memory outcomes, TyG was associated with lower CERAD delayed recall z-scores (*β* = −0.29; 95% CI, −0.52 to −0.06; *p* = 0.012), whereas associations with SNSB-II memory z-scores did not reach statistical significance (*β* = −0.31; 95% CI, −0.68 to 0.05; *p* = 0.087). The AST/ALT ratio was not significantly associated with memory domain measures (Table 2; Figure 2).

In the amyloid PET subset, neither TyG nor AST/ALT ratio was significantly associated with global amyloid PET SUVR (TyG: *β* = −0.03; 95% CI, −0.09 to 0.03; *p* = 0.30; AST/ALT: *β* = −0.02; 95% CI, −0.08 to 0.04; *p* = 0.57). APOE ε4 dose–modified associations were additionally evaluated in interaction models for TyG and AST/ALT ratio; while most interaction terms were not statistically significant, the TyG × APOE ε4 dose interaction for amyloid PET SUVR showed a borderline signal (*p* = 0.072) (Supplementary Table S4).

### 3.3 Interaction between fibrosis-4 stage and apolipoprotein E ε4 on global cognition and memory

When FIB-4 stage and APOE ε4 were modeled jointly with their interaction, the association between APOE ε4 and cognitive outcomes depended on FIB-4 stage (Table 3). Within the low FIB-4 stage, heterozygotes (*vs.* non-carriers) showed higher MMSE scores (*β* = 3.47; 95% CI, 0.81 to 6.12; *p* = 0.011), whereas the corresponding association for homozygotes (*vs.* non-carriers) was not statistically significant (*β* = 2.23; 95% CI, −1.29 to 5.75; *p* = 0.213). For visuospatial outcomes, a positive association at low FIB-4 was evident for CERAD visuospatial z-scores in heterozygotes (*β* = 3.38; 95% CI, 1.38 to 5.39; *p* = 0.001), whereas no association was observed for SNSB-II visuospatial z-scores (*β* = −0.06; 95% CI, −1.35 to 1.23; *p* = 0.929).

**Table 3:** Associations of FIB-4 stage and APOE ε4 dose with cognitive outcomes and amyloid PET SUVR: age-adjusted interaction models.

Characteristic	MMSE		SNSB visuospatial		CERAD visuospatial		Amyloid PET SUVR	
	*β* (95% CI)	*p*-value	*β* (95% CI)	*p*-value	*β* (95% CI)	*p*-value	*β* (95% CI)	*p*-value
Dose 1 vs 0 (low FIB-4)	3.47 (0.81, 6.12)	0.011*	−0.059 (−1.351, 1.234)	0.929	3.383 (1.378, 5.388)	0.001**	0.080 (−0.081, 0.241)	0.330
Dose 2 vs 0 (low FIB-4)	2.23 (−1.29, 5.75)	0.213	0.256 (−1.460, 1.972)	0.770	2.291 (−0.540, 5.123)	0.112	0.147 (−0.062, 0.357)	0.166
Intermediate × dose1	−4.80 (−7.75, −1.85)	0.001**	0.051 (−1.407, 1.509)	0.945	−3.307 (−5.496, −1.118)	0.003**	0.040 (−0.138, 0.218)	0.661
High × dose1	−4.26 (−7.74, −0.78)	0.017*	0.670 (−1.086, 2.425)	0.454	−2.969 (−5.462, −0.476)	0.020*	0.160 (−0.048, 0.368)	0.132
Intermediate × dose2	−4.54 (−8.52, −0.55)	0.026*	−0.449 (−2.395, 1.496)	0.650	−2.092 (−5.241, 1.058)	0.192	0.046 (−0.191, 0.283)	0.703
High × dose2	−4.62 (−9.28, 0.04)	0.052	0.851 (−1.602, 3.303)	0.496	−0.633 (−4.090, 2.824)	0.718	0.205 (−0.070, 0.481)	0.144

CI, Confidence interval. *means <0.05, **means <0.001.

Dose 0: non-carrier; 1: heterozygote; 2: homozygote.

Models adjusted for age, sex, education, hypertension, diabetes, and dyslipidemia.

Reference category: low FIB-4 and dose 0.

**Figure 3:**
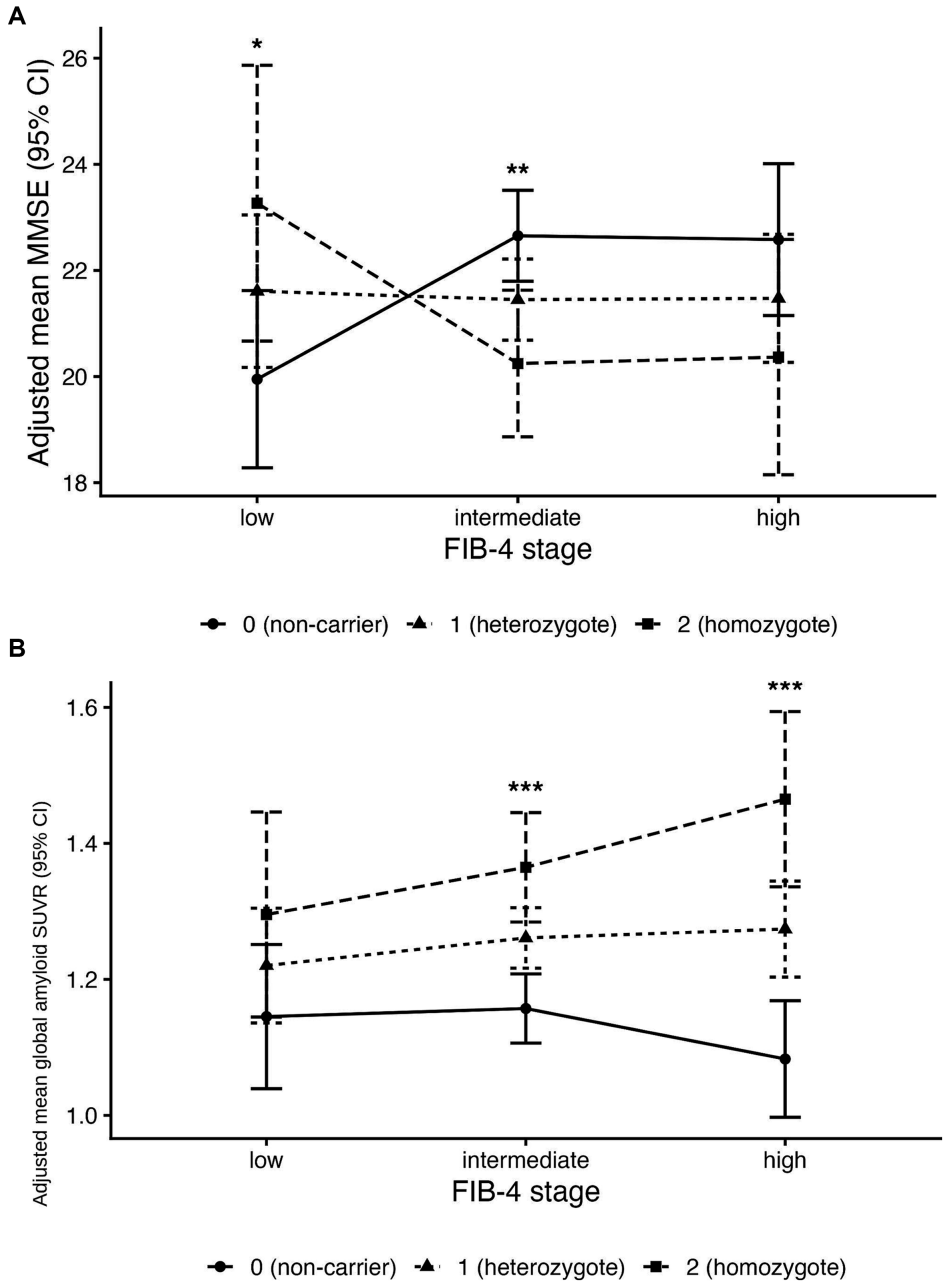
Interactive effects of FIB-4 stage and *APOE* ε4 genotype group on global cognition and cortical amyloid burden. **(A)** Adjusted estimated marginal means (EMMs) of MMSE scores across FIB-4 stages (low, intermediate, high) by *APOE* ε4 genotype group (non-carrier, heterozygote, homozygote). **(B)** Adjusted EMMs of global amyloid PET SUVR across FIB-4 stages by *APOE* ε4 genotype group. Error bars represent 95% confidence intervals. Models were adjusted for age_at_dx, sex, education, hypertension, diabetes, and dyslipidemia. FIB-4, Fibrosis-4 index; MMSE, Mini-Mental State Examination; SUVR, standardized uptake value ratio; *APOE*, apolipoprotein E.

Interaction terms indicated a stage-dependent attenuation and reversal of APOE ε4–associated cognitive differences at higher FIB-4 stages. In the MMSE model, relative to low FIB-4, the heterozygote-associated difference was lower in the intermediate stage (*β* for intermediate × heterozygote = −4.80; 95% CI, −7.75 to −1.85; *p* = 0.001) and in the high stage (*β* for high × heterozygote = −4.26; 95% CI, −7.74 to −0.78; *p* = 0.017). Additional interaction effects for homozygotes showed a similar direction (intermediate × homozygote: *β* = −4.54; 95% CI, −8.52 to −0.55; *p* = 0.026; high × homozygote: *β* = −4.62; 95% CI, −9.28 to 0.04; *p* = 0.052). These interaction patterns were consistent with the EMM plots across FIB-4 stages by APOE ε4 genotype groups (Figure 3A).

For CERAD visuospatial performance, interaction terms for heterozygotes were also negative and statistically significant (intermediate × heterozygote: *β* = −3.31; 95% CI, −5.50 to −1.12; *p* = 0.003; high × heterozygote: *β* = −2.97; 95% CI, −5.46 to −0.48; *p* = 0.020). Interaction terms for SNSB-II visuospatial z-scores were not significant (Table 3).

### 3.4 Fibrosis-4 staging, apolipoprotein E ε4 status, and cortical amyloid burden

In the amyloid PET subset, multivariable linear regression models were fitted to examine associations of FIB-4 stage and APOE ε4 genotype with global cortical amyloid PET SUVR (Table 3). Within the low FIB-4 stage (reference group), APOE ε4 heterozygotes and homozygotes did not show statistically significant differences in SUVR compared with non-carriers (heterozygote vs. non-carrier: *β* = 0.080; 95% CI, −0.081 to 0.241; *p* = 0.330; homozygote vs. non-carrier: *β* = 0.147; 95% CI, −0.062 to 0.357; *p* = 0.166).

Interaction terms suggested a stronger APOE ε4–associated increase in SUVR at higher FIB-4 stages. Relative to low FIB-4, the additional interaction effects were positive for both intermediate and high stages (intermediate × heterozygote: *β* = 0.040; 95% CI, −0.138 to 0.218; *p* = 0.661; high × heterozygote: *β* = 0.160; 95% CI, −0.048 to 0.368; *p* = 0.132; intermediate × homozygote: *β* = 0.046; 95% CI, −0.191 to 0.283; *p* = 0.703; high × homozygote: *β* = 0.205; 95% CI, −0.070 to 0.481; *p* = 0.144). Consistent with these coefficients, EMM plots showed relatively similar SUVR across APOE ε4 groups at low FIB-4, with a tendency toward higher SUVR with increasing ε4 dose at the high FIB-4 stage (Figure 3B).

As a sensitivity analysis, we refitted the interaction models without age adjustment. The overall pattern—attenuation and reversal of APOE ε4–associated differences in cognition across higher FIB-4 stages and the corresponding pattern for amyloid SUVR—remained qualitatively similar, supporting the robustness of the main findings (Supplementary Table S5).

### 3.5 Sensitivity analyses by cognitive diagnostic group

To evaluate whether the main patterns differed by cognitive diagnosis in this dementia-enriched registry, we performed sensitivity analyses stratified by a two-level diagnostic classification (Non-Dementia vs. Dementia). In the non-dementia group, several APOE ε4 group contrasts across FIB-4 stages were associated with higher amyloid PET SUVR estimates, including comparisons at the low FIB-4 stage and the high FIB-4 × homozygote contrast (Supplementary Table S2). In the Dementia group, corresponding contrast estimates were less consistent (Supplementary Table S2). An additional exploratory analysis using a three-level diagnostic classification (Normal/MCI/Dementia) showed significant associations in selected contrasts—most notably, higher SUVR among ε4 heterozygotes and homozygotes versus non-carriers at low FIB-4 within the MCI group (Supplementary Table S3)—while the distribution of participants across diagnosis-by-genotype categories was imbalanced.

## 4 Discussion

In this dementia trial-ready cohort, we examined how liver–metabolic stress markers and APOE ε4 genotype relate to late-life cognition and cortical amyloid burden. Three principal findings emerged. First, higher TyG index and AST/ALT ratio were associated with poorer global cognition and memory performance, whereas neither marker showed a clear association with cortical amyloid PET SUVR. Second, the association between FIB-4 stage and cognition differed by APOE ε4 genotype group, with a cross-over pattern across fibrosis stages for MMSE and CERAD visuospatial performance. Third, in the amyloid PET subset, higher FIB-4 stage was accompanied by greater separation in amyloid PET SUVR across APOE ε4 genotype groups, consistent with effect modification at higher fibrosis burden.

The associations of TyG index and AST/ALT ratio with global and memory-specific cognition, in the absence of clear relationships with amyloid PET SUVR in our data, are consistent with the possibility that insulin resistance and hepatic injury may influence cognition partly through non-amyloid pathways, for example, via systemic inflammation, microvascular damage, and impaired neurovascular coupling, as suggested by prior studies (Wang et al., 2023; Hong et al., 2021; Zhang Y.-L. et al., 2024; Vinuesa et al., 2021). Previous research has linked a higher TyG index to an increased risk of cognitive impairment and dementia in older adults (Wang et al., 2023; Hong et al., 2021). Our findings extend these observations by demonstrating that the TyG index is related not only to global MMSE scores but also to standardized memory z-scores across two independent neuropsychological batteries, even after adjusting for classical vascular risk factors. Similarly, a higher AST/ALT ratio was associated with lower MMSE scores, whereas associations with memory measures were not statistically significant (Zhang Y.-L. et al., 2024). Collectively, these patterns are compatible with the possibility that insulin resistance and hepatic injury contribute to cognitive vulnerability through predominantly non-amyloid pathways—such as systemic inflammation, endothelial dysfunction, and microvascular injury—rather than through measurable increases in cortical amyloid burden in this cross-sectional dataset (Vinuesa et al., 2021).

By contrast, the interaction between FIB-4 stage and APOE ε4 dose suggests that the association between ε4 allele burden and cognition varies systematically across the fibrosis spectrum. With low FIB-4 as the reference, ε4 heterozygosity (*vs.* non-carrier status) was associated with higher MMSE and CERAD visuospatial scores at low FIB-4, whereas this pattern reversed at higher fibrosis stages. Specifically, the ε4 dose–associated differences in MMSE were more than four points lower in the intermediate and high FIB-4 stages than in the low stage, with comparable negative interactions observed for CERAD visuospatial performance. Consistent with these findings, adjusted EMMs showed similar cognitive performance across ε4 groups at low FIB-4, but lower MMSE and visuospatial scores at intermediate and high FIB-4 stages among groups with higher ε4 burden. This cross-over pattern is consistent with effect modification, whereby liver–metabolic stress as indexed by FIB-4 stage is associated with greater cognitive impairment in an APOE ε4 genotype–dependent manner (Parikh et al., 2022; Weinstein et al., 2024b; Weinstein et al., 2024a; Rhea et al., 2020; Wang et al., 2021). However, several alternative explanations warrant consideration. The trial-ready registry design may introduce selection bias, as individuals with both high fibrosis burden and ε4 carriage who remain cognitively intact may be less likely to be enrolled, potentially inflating observed differences at higher FIB-4 stages. The cross-sectional design precludes causal inference, and the possibility of collider bias or residual confounding by cognitive diagnosis cannot be excluded. Accordingly, these results are interpreted as evidence of effect modification rather than causal or mechanistic effects.

In the amyloid PET subset, FIB-4 stage appeared to modify the association between APOE ε4 dose and cortical amyloid PET SUVR. In fully adjusted models, ε4 dose–related differences in SUVR were not prominent at low FIB-4; however, at high FIB-4, adjusted estimates tended to diverge across ε4 dose groups, with higher SUVR observed with greater ε4 allele burden (Table 3; Figure 3B). This interaction pattern suggests that liver-metabolic stress, as indexed by FIB-4 stage, is associated with greater amyloid burden in an ε4 dose-dependent manner, potentially reflecting impaired systemic amyloid clearance or a hepatic fibrosis–driven inflammatory milieu that favors cerebral amyloid accumulation (Weinstein et al., 2022; Peng et al., 2024; Parikh et al., 2022; Vinuesa et al., 2021; Weinstein et al., 2024a).

Taken together, these findings support an association between liver–metabolic stress markers and APOE ε4 with late-life cognitive performance and amyloid PET SUVR in this trial-ready cohort. The TyG index and AST/ALT ratio were associated with cognitive performance, whereas FIB-4 stage showed effect modification by APOE ε4 dose in relation to both cognition and amyloid PET SUVR. These results suggest that routinely available liver–metabolic indices may provide complementary information beyond conventional Alzheimer biomarkers and APOE genotyping in characterizing heterogeneity within dementia-enriched registries. From a trial-enrichment perspective, incorporating liver–metabolic indices alongside APOE ε4 genotype and amyloid PET may help generate testable hypotheses for multimodal risk models, which should be evaluated in longitudinal studies before clinical application.

**Figure 4:**
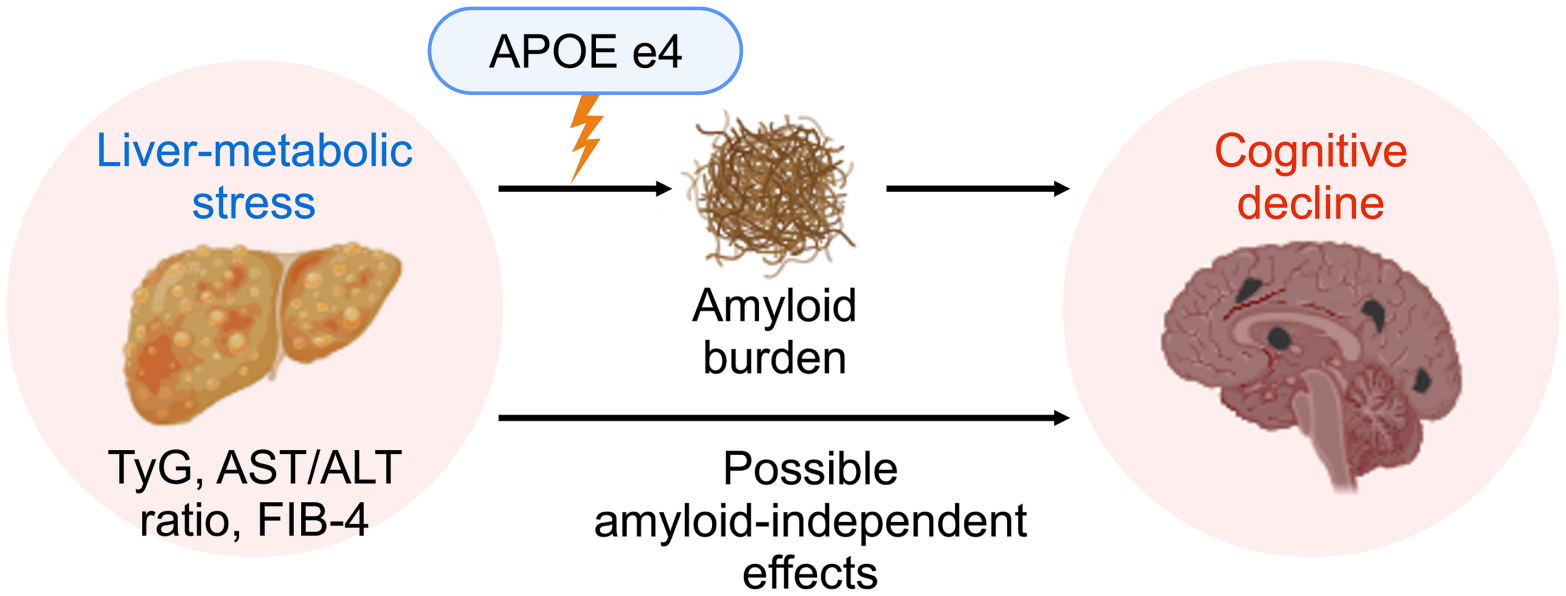
Conceptual schematic of liver–metabolic stress pathways associated with cognitive vulnerability and amyloid PET burden. The diagram summarizes hypothesized pathways linking liver–metabolic stress to cognitive vulnerability. TyG index and AST/ALT ratio were associated with cognitive performance in the absence of clear associations with amyloid PET SUVR, consistent with predominantly non-amyloid contributions. In contrast, FIB-4 stage showed effect modification by *APOE* ε4 genotype group in relation to cognition and amyloid PET SUVR. This schematic is provided for conceptual illustration and does not imply causal direction. TyG, triglyceride–glucose index; AST, aspartate aminotransferase; ALT, alanine aminotransferase; FIB-4, Fibrosis-4 index; *APOE*, apolipoprotein E; SUVR, standardized uptake value ratio.

Mechanistically, our findings are broadly consistent with the notion that liver–metabolic stress may influence Alzheimer-related risk through more than one pathway (Wu et al., 2025) (Figure 4). Experimental and imaging studies indicate that the liver contributes to peripheral clearance of circulating amyloid-*β* via low-density lipoprotein receptor–related pathways and other hepatic uptake systems, and that impaired liver function or fibrosis may reduce this clearance capacity (Garcia et al., 2022; Maarouf et al., 2018). In this context, the greater separation in cortical amyloid PET SUVR across APOE ε4 groups at high FIB-4 stages is consistent with prior hypotheses linking hepatic fibrosis to altered amyloid homeostasis in genetically susceptible individuals (Peng et al., 2024; Wu et al., 2025). Separately, TyG index and AST/ALT ratio were associated with global and memory-specific cognition, findings that align with models implicating insulin resistance, endothelial dysfunction, and cerebral small-vessel disease in neurovascular and synaptic vulnerability (Teng et al., 2022; Yang et al., 2019). Whether these associations reflect pathways that are truly independent of amyloid accumulation cannot be determined from the present cross-sectional data. The preferential involvement of episodic memory and visuospatial performance across two independent neuropsychological batteries further suggests vulnerability of hippocampal and posterior parietal–occipital networks, which are sensitive to both Alzheimer pathology and vascular–metabolic injury (Ilardi et al., 2022; Plaza-Rosales et al., 2023; Zhao et al., 2021).

We conducted several sensitivity analyses to evaluate the robustness of the main patterns. First, because FIB-4 incorporates age as one of its components, interaction models were refitted without age adjustment to assess potential collinearity; the overall interaction patterns remained qualitatively similar across cognitive and amyloid PET outcomes (Supplementary Table S5). The consistency of findings with and without age adjustment suggesting that the observed patterns are not solely attributable to the age component embedded in the FIB-4 formula. Second, to address potential confounding by cognitive diagnosis in this dementia-enriched registry, we performed diagnosis-stratified sensitivity analyses at two levels (non-dementia *vs.* dementia) and three levels (cognitively normal, MCI, and dementia), which did not materially change the overall interpretation of the main patterns (Supplementary Tables S2, S3).

Several sources of confounding and reverse causation warrant consideration. Dementia-related metabolic changes, including weight loss, reduced physical activity, and altered dietary intake, could independently modify liver enzyme levels and FIB-4 values, raising the possibility that observed associations partly reflect disease-related metabolic consequences rather than antecedent hepatic pathology (Singh-Manoux et al., 2018; Wagner et al., 2018). Additionally, commonly used medications such as statins and metformin exert direct effects on liver enzyme levels and insulin sensitivity that may influence TyG index, AST/ALT, and FIB-4 values beyond binary comorbidity adjustment (Ma et al., 2024; Hu et al., 2021).

From both clinical and research perspectives, our findings suggest that simple liver–metabolic indices may provide complementary information to conventional Alzheimer biomarkers and genetic profiling. The TyG index and AST/ALT ratio were associated with global and memory-specific cognition even after adjustment for vascular risk factors and APOE ε4, in the absence of clear associations with amyloid PET SUVR in this study, which is compatible with non-amyloid contributions to cognitive vulnerability. In parallel, the combination of FIB-4 stage and APOE ε4 dose revealed that higher fibrosis burden was associated with greater divergence in cognitive performance and amyloid PET SUVR across APOE ε4 dose groups. In trial-ready cohorts such as DPK-TRR, incorporating liver–metabolic markers alongside APOE ε4 genotype and amyloid PET may help generate testable hypotheses for enrichment and risk stratification models, which should be evaluated in longitudinal studies before clinical application.

This study has several strengths. We leveraged a relatively large, deeply phenotype, dementia trial-ready cohort with harmonized clinical, laboratory, and neuropsychological assessments, and we examined liver–metabolic markers, APOE ε4 dose, domain-specific cognition, and amyloid PET burden within the same framework. By evaluating the TyG index, AST/ALT ratio, and FIB-4 stage using consistent covariate adjustment and convergent cognitive indices from both the SNSB-II and CERAD-K batteries, we were able to contrast associations with cognition that were not accompanied by clear amyloid PET differences with interaction patterns in which FIB-4 stage modified the relationship between APOE ε4 dose and both cognitive performance and amyloid PET SUVR.

Several limitations should be acknowledged. First, the cross-sectional design precludes causal inference regarding temporal relationships among liver–metabolic stress, amyloid accumulation, and cognitive decline; longitudinal follow-up will be required to determine whether change in TyG index, FIB-4 stage, or related markers predicts subsequent cognitive deterioration or conversion to dementia. Second, the FIB-4 index and AST/ALT ratio are indirect, noninvasive surrogates rather than histological measures, and misclassification is possible, particularly in older adults with comorbidities. Third, because FIB-4 incorporates age as a component and age was also included as a covariate, some degree of collinearity is inherent; although sensitivity analyses without age adjustment yielded qualitatively consistent findings, FIB-4 associations should be interpreted as reflecting a composite index rather than an age-independent fibrosis signal. Fourth, the DPK-TRR is a trial-ready registry enriched for cognitive impairment, which may limit generalizability and introduce selection effects that could influence observed interactions, particularly at higher FIB-4 stages where sample sizes were smaller. Fifth, although we adjusted for major vascular risk factors and performed diagnosis-stratified sensitivity analyses, residual confounding by unmeasured metabolic, lifestyle, medication-related, or cerebrovascular factors cannot be excluded. Reverse causation cannot be excluded, as dementia-related neurodegeneration may alter systemic metabolic homeostasis and thereby change liver-metabolic marker values, a concern that has been specifically raised in recent longitudinal studies of MASLD and dementia risk (Bartholdy et al., 2026). Sixth, amyloid PET analyses combined data from two tracers and lacked tau PET and detailed small-vessel disease markers, limiting mechanistic inference regarding the specific neurodegenerative or vascular pathways involved. Finally, interaction estimates—particularly within high FIB-4 categories and among ε4 homozygotes—may be imprecise given the relatively small subgroup sizes, and replication in larger independent cohorts is needed before these findings can inform clinical practice.

In summary, this study supports an association between liver–metabolic stress markers and APOE ε4 genotype with late-life cognitive performance and amyloid PET SUVR in a dementia trial-ready registry. Routine markers of insulin resistance and hepatic injury (TyG index and AST/ALT ratio) were associated with global and memory-specific cognitive performance in the absence of clear associations with amyloid PET SUVR, whereas FIB-4 stage showed effect modification by APOE ε4 in relation to both cognition and amyloid PET signal. These findings suggest that liver–metabolic indices may provide complementary information beyond traditional Alzheimer biomarkers and APOE genotyping in characterizing heterogeneity within dementia-enriched cohorts. Future longitudinal and mechanistic studies, incorporating more granular imaging of vascular and neurodegenerative changes, are warranted to evaluate temporal relationships and to test whether improving liver and metabolic health modifies cognitive trajectories, particularly in individuals with higher fibrosis burden and greater ε4 allele burden.

## Data Availability

Publicly available datasets were analyzed in this study. This data can be found here: The data that support the findings of this study are available from the Dementia Platform Korea (DPK) at https://www.dpk.re.kr. Access to these data is subject to registration and approval by the DPK.
