# Supplementary material for: Liver–metabolic stress, apolipoprotein E ε4, and cognition and amyloid burden: findings from the dementia platform Korea trial-ready registry
Source: Front Aging Neurosci. 2026 Mar 11;18:1773977. doi: 10.3389/fnagi.2026.1773977 (PMC13012996; doi:10.3389/fnagi.2026.1773977)
Supplement: Supplementary file 1 [file Data_Sheet_1.zip › Table S1.docx]

**Supplementary Table S1. Distribution of APOE ε4 dose across FIB-4 stages**

(A) Main sample

| fib4_stage | 0 (non-carrier) | 1 (heterozygote) | 2 (homozygote) |
| --- | --- | --- | --- |
| Low | 47 (50.5%) | 32 (34.4%) | 14 (15.1%) |
| intermediate | 183 (50.1%) | 136 (37.3%) | 46 (12.6%) |
| High | 61 (49.6%) | 45 (36.6%) | 17 (13.8%) |

(B) PET subset

| dataset | fib4_stage | 0 (non-carrier) | 1 (heterozygote) | 2 (homozygote) |
| --- | --- | --- | --- | --- |
| PET subset | low | 33 (45.2%) | 28 (38.4%) | 12 (16.4%) |
| PET subset | intermediate | 159 (49.2%) | 123 (38.1%) | 41 (12.7%) |
| PET subset | high | 53 (48.6%) | 40 (36.7%) | 16 (14.7%) |
