# Supplementary material for: Liver–metabolic stress, apolipoprotein E ε4, and cognition and amyloid burden: findings from the dementia platform Korea trial-ready registry
Source: Front Aging Neurosci. 2026 Mar 11;18:1773977. doi: 10.3389/fnagi.2026.1773977 (PMC13012996; doi:10.3389/fnagi.2026.1773977)
Supplement: Supplementary file 1 [file Data_Sheet_1.zip › Table S2.docx]

| Diagnosis | Characteristic | MMSE | | Amyloid PET SUVR | |
| --- | --- | --- | --- | --- | --- |
|  |  | β (95% CI) | p-value | β (95% CI) | p-value |
| NonDementia | Dose 1 vs 0 (low FIB-4) | 0.05 (-2.06, 2.16) | 0.961 | 0.203 (0.013, 0.393) | 0.036* |
| NonDementia | Dose 2 vs 0 (low FIB-4) | -0.01 (-2.73, 2.71) | 0.994 | 0.227 (0.005, 0.449) | 0.045* |
| NonDementia | High × dose2 | -2.25 (-6.08, 1.58) | 0.250 | 0.331 (0.029, 0.634) | 0.032* |
| Dementia | Dose 1 vs 0 (low FIB-4) | 1.86 (-1.99, 5.71) | 0.343 | -0.196 (-0.481, 0.090) | 0.178 |
| Dementia | Dose 2 vs 0 (low FIB-4) | 3.50 (-1.88, 8.87) | 0.200 | -0.071 (-0.472, 0.330) | 0.728 |
| Dementia | Intermediate × dose2 | -0.92 (-7.60, 5.76) | 0.787 | 0.559 (0.043, 1.074) | 0.034* |
| Dementia | High × dose2 | -4.73 (-12.45, 2.99) | 0.229 | 0.554 (-0.032, 1.141) | 0.064 |

**Supplementary Table S2. Diagnosis-stratified (dx2: NonDementia vs Dementia) sensitivity analyses highlighting APOE ε4 dose contrasts within FIB-4 stages for amyloid PET SUVR**
