# Supplementary material for: Liver–metabolic stress, apolipoprotein E ε4, and cognition and amyloid burden: findings from the dementia platform Korea trial-ready registry
Source: Front Aging Neurosci. 2026 Mar 11;18:1773977. doi: 10.3389/fnagi.2026.1773977 (PMC13012996; doi:10.3389/fnagi.2026.1773977)
Supplement: Supplementary file 1 [file Data_Sheet_1.zip › Table S3.docx]

**Supplementary Table S3. Exploratory analyses stratified by 3-level cognitive diagnosis (normal/MCI/dementia): APOE ε4 dose contrasts across FIB-4 stages for MMSE and amyloid PET SUVR**

| Diagnosis | Characteristic | MMSE global | | Amyloid PET SUVR | |
| --- | --- | --- | --- | --- | --- |
|  |  | β (95% CI) | p-value | β (95% CI) | p-value |
| Normal | APOE ε4 dose 1 (vs 0) [at low FIB-4] | 2.89 (-3.53, 9.31) | 0.368 | 0.371 (-0.377, 1.119) | 0.319 |
| Normal | APOE ε4 dose 2 (vs 0) [at low FIB-4] | 5.66 (-1.33, 12.66) | 0.110 | -0.489 (-1.265, 0.288) | 0.208 |
| Normal | Intermediate × dose1 (additional vs low) | -4.98 (-12.05, 2.09) | 0.162 | -0.470 (-1.303, 0.363) | 0.258 |
| Normal | High × dose1 (additional vs low) | -5.66 (-13.14, 1.82) | 0.134 | -0.167 (-1.066, 0.733) | 0.707 |
| Normal | Intermediate × dose2 (additional vs low) | -9.60 (-17.87, -1.33) | 0.024* | 0.586 (-0.357, 1.530) | 0.214 |
| Normal | High × dose2 (additional vs low) | -4.66 (-13.77, 4.45) | 0.307 | 0.730 (-0.261, 1.721) | 0.143 |
| MCI | APOE ε4 dose 1 (vs 0) [at low FIB-4] | -0.47 (-3.02, 2.08) | 0.716 | 0.232 (0.023, 0.440) | 0.030* |
| MCI | APOE ε4 dose 2 (vs 0) [at low FIB-4] | -1.04 (-4.23, 2.15) | 0.521 | 0.308 (0.068, 0.548) | 0.012* |
| MCI | Intermediate × dose1 (additional vs low) | -0.82 (-3.66, 2.02) | 0.570 | -0.155 (-0.386, 0.076) | 0.187 |
| MCI | High × dose1 (additional vs low) | -0.32 (-3.83, 3.19) | 0.859 | 0.061 (-0.226, 0.348) | 0.676 |
| MCI | Intermediate × dose2 (additional vs low) | -0.88 (-4.59, 2.84) | 0.641 | 0.005 (-0.278, 0.288) | 0.973 |
| MCI | High × dose2 (additional vs low) | -4.03 (-9.72, 1.65) | 0.163 | 0.049 (-0.400, 0.498) | 0.830 |
| Dementia | APOE ε4 dose 1 (vs 0) [at low FIB-4] | 3.91 (0.30, 7.52) | 0.034* | -0.002 (-0.263, 0.258) | 0.985 |
| Dementia | APOE ε4 dose 2 (vs 0) [at low FIB-4] | 1.55 (-3.37, 6.47) | 0.536 | 0.093 (-0.327, 0.514) | 0.662 |
| Dementia | Intermediate × dose1 (additional vs low) | -4.41 (-8.36, -0.45) | 0.029* | 0.187 (-0.094, 0.469) | 0.191 |
| Dementia | High × dose1 (additional vs low) | -6.06 (-10.61, -1.50) | 0.009** | 0.311 (-0.013, 0.635) | 0.060 |
| Dementia | Intermediate × dose2 (additional vs low) | -1.96 (-7.37, 3.44) | 0.475 | 0.022 (-0.428, 0.471) | 0.924 |
| Dementia | High × dose2 (additional vs low) | -1.65 (-7.55, 4.25) | 0.582 | 0.222 (-0.256, 0.699) | 0.362 |
