# Supplementary material for: Liver–metabolic stress, apolipoprotein E ε4, and cognition and amyloid burden: findings from the dementia platform Korea trial-ready registry
Source: Front Aging Neurosci. 2026 Mar 11;18:1773977. doi: 10.3389/fnagi.2026.1773977 (PMC13012996; doi:10.3389/fnagi.2026.1773977)
Supplement: Supplementary file 1 [file Data_Sheet_1.zip › Table S4.docx]

**Supplementary Table S4. Interaction models of TyG index, AST/ALT ratio and APOE ε4 dose for cognitive outcomes and amyloid PET SUVR**

| Outcome | Exposure | Effect | Estimate β (95% CI) | p-value |
| --- | --- | --- | --- | --- |
| MMSE global | TyG | APOE ε4 dose (per +1 allele) | 4.950 [-7.308, 17.209] | 0.428 |
|  |  | APOE ε4 dose × TyG index | -0.650 [-2.065, 0.764] | 0.367 |
| Amyloid PET SUVR | TyG | APOE ε4 dose (per +1 allele) | -0.535 [-1.249, 0.178] | 0.141 |
|  |  | APOE ε4 dose × TyG index | 0.075 [-0.007, 0.158] | 0.072 |
| MMSE global | AST/ALT | APOE ε4 dose (per +1 allele) | -0.628 [-2.680, 1.424] | 0.548 |
|  |  | APOE ε4 dose × AST/ALT ratio | -0.003 [-1.372, 1.367] | 0.997 |
| Amyloid PET SUVR | AST/ALT | APOE ε4 dose (per +1 allele) | 0.101 [-0.023, 0.225] | 0.110 |
|  |  | APOE ε4 dose × AST/ALT ratio | 0.013 [-0.072, 0.097] | 0.767 |

Adjusted for age_at_dx, sex, education, HTN, DM, and dyslipidemia; APOE ε4 dose coded as 0/1/2 (allele count).

* means <0.05
