# Supplementary material for: Liver–metabolic stress, apolipoprotein E ε4, and cognition and amyloid burden: findings from the dementia platform Korea trial-ready registry
Source: Front Aging Neurosci. 2026 Mar 11;18:1773977. doi: 10.3389/fnagi.2026.1773977 (PMC13012996; doi:10.3389/fnagi.2026.1773977)
Supplement: Supplementary file 1 [file Data_Sheet_1.zip › Table S5.docx]

| **Supplement Table S5. Sensitivity analysis without age adjustment: Associations of FIB-4 stage and APOE ε4 dose with cognitive outcomes and amyloid PET SUVR (interaction models)** |
| --- |
| \| Characteristic \| MMSE \| \| \| SNSB Visuospatial \| \| \| CERAD Visuospatial \| \| Amyloid PET SUVR \| \| \| --- \| --- \| --- \| --- \| --- \| --- \| --- \| --- \| --- \| --- \| --- \| \| β (95% CI) \| \| p-value \| β (95% CI) \| p-value \| \| β (95% CI) \| p-value \| β (95% CI) \| p-value \| \| Dose 1 vs 0 (low FIB-4) \| 1.34 (-1.11, 3.78) \| 0.283 \| \| -0.252 (-1.427, 0.922) \| \| 0.674 \| 1.594 (-0.229, 3.418) \| 0.087 \| 0.072 (-0.076, 0.220) \| 0.340 \| \| Dose 2 vs 0 (low FIB-4) \| 0.09 (-3.17, 3.35) \| 0.956 \| \| 0.123 (-1.442, 1.687) \| \| 0.878 \| 0.321 (-2.241, 2.882) \| 0.806 \| 0.145 (-0.053, 0.343) \| 0.150 \| \| Intermediate × dose1 \| -5.19 (-8.16, -2.22) \| 0.001** \| \| 0.108 (-1.210, 1.426) \| \| 0.872 \| -3.310 (-5.551, -1.068) \| 0.004** \| 0.042 (-0.137, 0.222) \| 0.646 \| \| High × dose1 \| -4.57 (-8.10, -1.03) \| 0.011* \| \| 0.662 (-0.924, 2.248) \| \| 0.412 \| -2.952 (-5.505, -0.399) \| 0.023* \| 0.163 (-0.046, 0.372) \| 0.127 \| \| Intermediate × dose2 \| -4.87 (-8.90, -0.85) \| 0.018* \| \| -0.317 (-2.112, 1.477) \| \| 0.729 \| -2.049 (-5.266, 1.167) \| 0.211 \| 0.049 (-0.189, 0.287) \| 0.686 \| \| High × dose2 \| -4.93 (-9.69, -0.17) \| 0.042* \| \| 0.875 (-1.399, 3.149) \| \| 0.450 \| -0.534 (-4.059, 2.991) \| 0.766 \| 0.209 (-0.068, 0.487) \| 0.139 \|   Abbreviation: CI = Confidence Interval * means <0.05, ** means <0.001  Dose 0: non-carrier; 1:heterozygote; 2:homozygote |

*Same model specification as Table 3A but without age (sensitivity analysis).*

Reference category: low FIB-4 & dose 0.
